# Genome-wide characterization and expression analysis of *bHLH* gene family in physic nut (*Jatropha curcas* L.)

**DOI:** 10.7717/peerj.13786

**Published:** 2022-08-09

**Authors:** Lin Zhang, Wei Chen, Rongrong Liu, Ben Shi, Youju Shu, Haoyu Zhang

**Affiliations:** School of Environmental Engineering and Chemistry, Luoyang Institute of Science and Technology, Luoyang, Henan, China

**Keywords:** Physic nut, bHLH transcription factor, Abiotic stress

## Abstract

The basic helix loop helix (bHLH) transcription factor perform essential roles in plant development and abiotic stress. Here, a total of 122 bHLH family members were identified from the physic nut (*Jatropha curcas* L.) genomic database. Chromosomal localization results showed that 120 members were located on 11 chromosomes. The phylogenetic tree manifested that the *JcbHLHs* could be grouped into 28 subfamilies. Syntenic analysis showed that there were 10 bHLH collinear genes among the physic nut, *Arabidopsis thaliana* and *Oryza sativa*. These genes, except *JcbHLH84*, were highly expressed in various tissues of the physic nut, implying a key role in plant development. Gene expression profiles showed that ten genes (especially *JcbHLH33*, *JcbHLH45* and *JcbHLH55*) correspond to both salinity and drought stresses; while eight genes only respond to salinity and another eight genes only respond to drought stress. Moreover, the protein interaction network revealed that the *JcbHLHs* are involved in growth, development and stress signal transduction pathways. These discoveries will help to excavate several key genes may involve in salt or drought stresses and seed development, elucidate the complex transcriptional regulation mechanism of *JcbHLH* genes and provide the theoretical basis for stress response and genetic improvement of physic nut.

## Introduction

The basic helix loop helix (bHLH) transcription factor, the second-largest gene family in plants, widely exists in eukaryotes (Feller et al., 2011). There are about 60 amino acids of the bHLH domain that consist of two different functional regions, one is the basic region and the other is the helix loop helix (HLH) region. The basic region is located at the N-terminal of the bHLH domain, which is composed of 13–17 major basic amino acids and combines with the motif CANNTG of E-box, is mainly related to DNA binding (Atchley, Terhalle & Dress, 1999). The HLH region is located at the C-terminal of the bHLH domain, containing both hydrophilic and lipophilic α-helices. Two α-helices are separated by different lengths of connecting loops, forming a helix loop helix structure. The interaction between the α-helix can form a homodimer or heterodimer, which can bind to diverse regions of the target gene promoters to mediate the gene transcription (Ellenberger et al., 1994; Heim et al., 2003; Toledo-Ortiz & Quail, 2003). Based on conserved regions, DNA binding patterns and phylogenetic relationships, bHLHs were divided into six major groups (groups A–F) (Ledent & Vervoort, 2001; Atchley & Fitch, 1997), which have been subdivided into several smaller orthologous subgroups (Simionato et al., 2007). The *bHLH* transcription factor gene family is generally grouped into 15-26 subfamilies in plants (Toledo-Ortiz & Quail, 2003); but if atypical bHLH proteins are included, they can be divided into more subfamilies (Carretero-Paulet et al., 2010).

In previous studies, more and more bHLH proteins have been systematically authenticated in plants, such as tomato (Sun, Fan & Ling, 2015), rice (Li et al., 2006), *Arabidopsis* (Bailey et al., 2003), Chinese cabbage (Song et al., 2014), *Zea mays* (Zhang et al., 2018a), pear (Dong et al., 2021), cucurbits (Xu et al., 2022), poplar and moss (Carretero-Paulet et al., 2010). Studies have shown that plant bHLH proteins play key roles in their growth (Massari & Murre, 2000; Steven et al., 2005), flowering time regulation (Aslam et al., 2020; Wang et al., 2017), secondary metabolites (Hu et al., 2016; Petroni & Cominelli, 2000; Wang et al., 2019a; Xu, Dubos & Lepiniec, 2015), stress response (Chen et al., 2015; Huang et al., 2013; Ke et al., 2017; Xi, Yu & Na, 2018; Zhang et al., 2020b), hormone signal transduction (Wang et al., 2016; Nakata et al., 2013), and so on. Subsequently, more and more functions of *bHLH* genes have been identified. For example, *AtPRE1* has been reported to involve in gibberellin-dependent response (Lee et al., 2006). *AtGL3* is essential for anthocyanin accumulation caused by nitrogen consumption (Feyissa et al., 2009). *TabHLH39* is associated with salt tolerance (Zhai et al., 2016). The MYB-bHLH-WD40 complex is considered to promote anthocyanins biosynthesis and plant growth (Li, 2014). Therefore, the bHLHs can work through forming homologous or heterologous complexes with other proteins.

Physic nut (*Jatropha curcas* L.), a deciduous perennial shrub of *Euphorbiaceae*, is one of the most important energy plants in the world. It has many agronomic advantages, such as a high yield of seed production, high oil content in seeds, and tolerance to various stresses (Divakara et al., 2010; King et al., 2009). The *bHLH* transcription factors regulate many key biological processes in plants, however, *bHLHs* in the physic nut have not been reported yet. The genome database of the physic nut and its global gene expression profiles under several abiotic stress conditions provided useful resources for searching critical genes related to biological functions at the genome level (Wu et al., 2015; Zhang et al., 2015; Zhang, 2014). A total of 122 putative *JcbHLH* genes in all were identified in this study, the phylogenetic relationships, chromosome distribution, gene structure and duplication, conserved motifs, syntenic analysis and protein-protein interaction network were conducted at a genome-wide level. Furthermore, the expression patterns of *JcbHLH* genes in various tissues and at different conditions were also performed. These findings will provide a valuable basis to elucidate the functions of the JcbHLH family in the process of growth and stress response of physical nuts and other crops.

## Materials & Methods

### Plant materials

The physic nut seed used in this research is cultivar GZQX0401. The seedling cultivation condition and methods of stress treatment were carried out according to our previous studies (Zhang et al., 2015; Zhang, 2014). Seeds germinated in sand and grown in a mixture of 3:1 sand and soil in light incubator (day/night: 14 h/10 h; daily temperature: 25–33°C). After the first true leaves appeared, irrigation was carried out every other day with Hoagland nutrient solution (pH 6.0). Seedlings were subjected to salt stress (adding 100 mM NaCl daily to Hoagland nutrient solution) and drought stress (stopping nutrient solution watering) at the six-leaf stage. The control group was given Hoagland nutrient solution every day.

### RNA isolation and expression analysis

Total RNA was extracted based on the modified CTAB method (Zhang, 2014). RNase-free DNase I (TIANGEN, Beijing, China) was used to remove the genomic DNA, and M-MLV reverse transcriptase (Promega, Madison, WI) was used to reversely transcribe the RNA into cDNA. The physic nut roots and leaves under salt and drought treatment were harvested for qRT-PCR analysis. Three biological replicates were performed for each treatment. The detailed information of primers was listed in Table S1. The gene expression profile raw data (SRA: PRJNA257901, PRJNA244896 and SRR2039597) were download from NCBI. Firstly, the data of sequence reads were processed by Trimmomatic and STAR software to remove the adaptor and the low-quality sequences, secondly, the clean reads were were mapped to the reference genome using the STAR software with default parameters. Finally, gene expression levels were calculated and normalized to the FPKM (fragments per kilo-bases per million) value. According to lg^(FPKMvalue+0.0001)^ conversion of the FPKMs, a heatmap of *JcbHLHs* expression in various tissues was constructed. In additon, the heatmap of JcbHLHs expression under salt and drought treatment was constructed based on the log_2_^(FPKMvalue+0.1)^ ratio of sample to control.

### Identification of JcbHLHs

To identify the bHLH family member in physic nut, the bHLH protein sequences of *Arabidopsis* and rice were used as query sequences to carry out BLASTP search against the physic nut genome (Wu et al., 2015). Sequences with e-value cut-off less than 1e^−10^ were selected as target genes. The bHLH protein sequences (Table S2) of *Arabidopsis* were downloaded from TAIR (http://www.arabidopsis.org/), while the sequences of rice (Table S2) were downloaded from the Plant Transcription Factor Database (http://planttfdb.gao-lab.org/). Furthermore, the PFAM database (http://pfam.xfam.org/) was used to download the hidden Markov model (HMM) file of the bHLH domain (PF00010) (Finn et al., 2016). Based on the raw bHLH HMM, the protein sequences from physic nut genome (GenBank accession number AFEW00000000) with *E*-value < 1.7e−10 were selected and verified by an intact bHLH domain. The HMMER v3 software (Finn, Clements & Eddy, 2011) was used to construct a physic nut-specific bHLH HMM, and then the protein sequences with an *E*-value lower than 0.001 were extracted. To verify the identified genes, the SMART (http://smart.embl-heidelberg.de/) and PFAM program was used to detect bHLH domains, and then removed the sequences lacking the bHLH domain. After alignment by Clustal X, the redundant sequences of the detected JcbHLHs were removed (Larkin et al., 2007). Finally, the parameters of the JcbHLH protein length, molecular weight and isoelectric point were predicted based on the online ExPasy program (http://www.expasy.org/tools/).

### Chromosomal location, gene structure and conserved motif study of JcbHLHs

According to the genome coordinates in physic nut, every *JcbHLH* was mapped to the genome. The map distance (cM, centiMorgans) was computed through the maximum likelihood mapping algorithm and Kosambi mapping function (Wu et al., 2015). The Map-Chart software package was used to constructed the linkage map of *JcbHLH*s (Voorrips, 2002).

The exon/intron structures and exons number of *JcbHLHs* were predicted by the website Gene Structure Display Server (GSDS, http://gsds.gao-lab.org/) (Gao et al., 2015). Conserved motifs were analyzed using the MEME Suite version 5.4.1 (http://meme-suite.org/tools/meme) (Bailey et al., 2015). All parameters are set to default values, except the number of different motifs is set to 10.

### Phylogenetic analysis of *JcbHLH* genes

The 122 JcbHLH protein sequences were used for multiple sequence alignment using clustalX1.83 software. According to the sequence alignment, the unrooted phylogenetic tree was built by MEGA 7 program with the maximum likelihood method and the bootstrap value of 1000 replicates and default parameters was adopted (Sudhir, Glen & Koichiro, 2016). The phylogenetic tree was plotted through the EvolView tool (http://www.evolgenius.info).

### The promoter analysis of *JcbHLH* genes

The 2-kb long sequences from the transcript start site of the 122 *JcbHLH*s were extracted based on the physic nut genome database (Wu et al., 2015). The PlantCARE (http://bioinformatics.psb.ugent.be/webtools/plantcare/html/) software (Lescot, 2002) was utilized to analyze the cis-elements on the promoter regions of these genes.

### Gene duplication and synteny analysis

The gene duplication events were analyzed through the Multiple Collinearity Scan toolkit (MCScanX) with the default parameters. To study the synteny relationship of the bHLHs in physic nut and other three selected species (*Arabidopsis*, rice and grape), the syntenic analysis maps were constructed by the MCScanX program package (Wang et al., 2012).

### Protein–protein interaction network prediction

The STRING website (https://string-db.org/) was utilized to speculate the protein-protein interactions among 122 JcbHLHs. The orthologs of *Arabidopsis thaliana* were chosen as references. Based on the whole step of the BLAST, an interaction network among *JcbHLHs* was constructed utilizing the highest score gene (bitscore).

## Results

### Identification of JcbHLHs

The bHLH family members of physic nut were identified and all candidate genes were validated to determine whether they had complete bHLH domains. 122 bHLH genes (Table S3) were detected in the physic nut genome (Wu et al., 2015), which were named from *JcbHLH1* to *JcbHLH122* based on their gene structure and motifs. The corresponding protein, gene IDs and the relevant information of identified *JcbHLHs* were listed in Table S3. The number of amino acids contained in JcbHLH protein ranged from 91 (JcbHLH19) to 829 (JcbHLH105). The molecular weight of the proteins was predicted between 10.5 kDa (JcbHLH19) to 90.3 kDa (JcbHLH105), and their predicted pIs (isoelectric points) were ranged from 4.62 (JcbHLH89) to 11.16(JcbHLH58). Depending on their physical and chemical properties, it is speculated that family members may have multiple functions. Physic nut contains fewer bHLH proteins than *Arabidopsis thaliana* (162) and rice (167) (Carretero-Paulet et al., 2010). One possible explanation is that the *JcbHLH* family genes have not experienced large-scale genome-wide doubling events (Wu et al., 2015). However, large-scale multiplication events led to the expansion of *bHLH* family genes in *Arabidopsis* and rice (Cannon et al., 2004; Carretero-Paulet et al., 2010).

### Phylogenetic analysis of JcbHLHs

**Figure 1:**
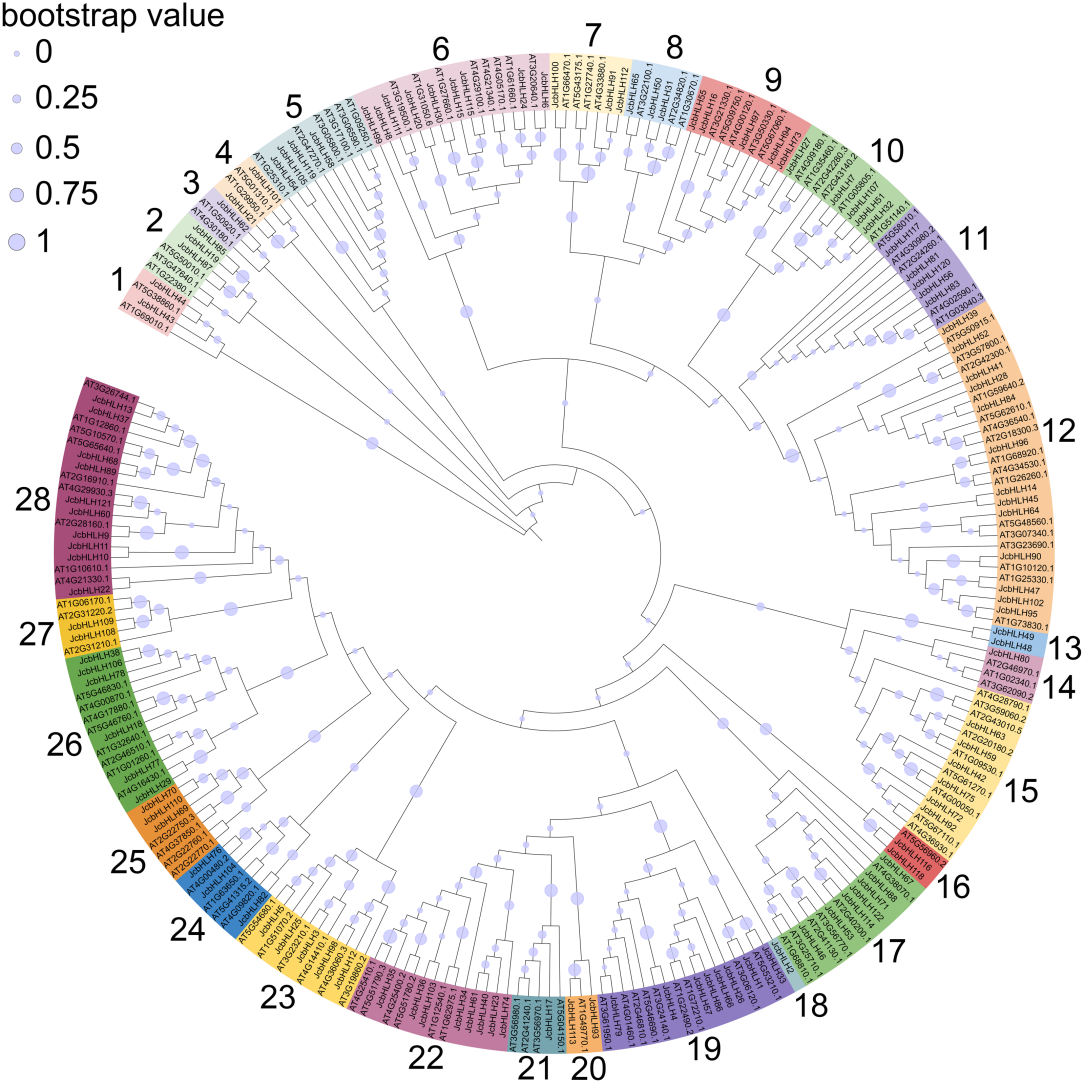
Phylogenetic tree construction (circle tree) and subfamily classifications of the JcbHLH and AtbHLH proteins. The 28 numbers represent each subfamily and are displayed in a different background color.

To explore the evolutionary relationship of the bHLH family, the phylogenetic tree of physic nut and *Arabidopsis* bHLH protein sequences was constructed *via* the maximum likelihood method (Fig. 1). The unrooted tree revealed that the JcbHLHs were classified into 28 subfamilies, which were named from 1 to 28, respectively. The number of bHLH members is different in each group, and group 12 has the largest number with 13 bHLH members. Unlike other clades, clade 3, 14, 18 and 21 contain individual bHLH protein, which means that JcbHLH62, JcbHLH80, JcbHLH2 and JcbHLH17 are unique. Except for clade 3, 14, 18 and 21, the number of genes per clade varies widely from 2 (clade 1, 4, 13, 16, 20 and 27) to 13 (clade 12).

### Conserved motif and the gene structure analysis of JcbHLHs

**Figure 2:**
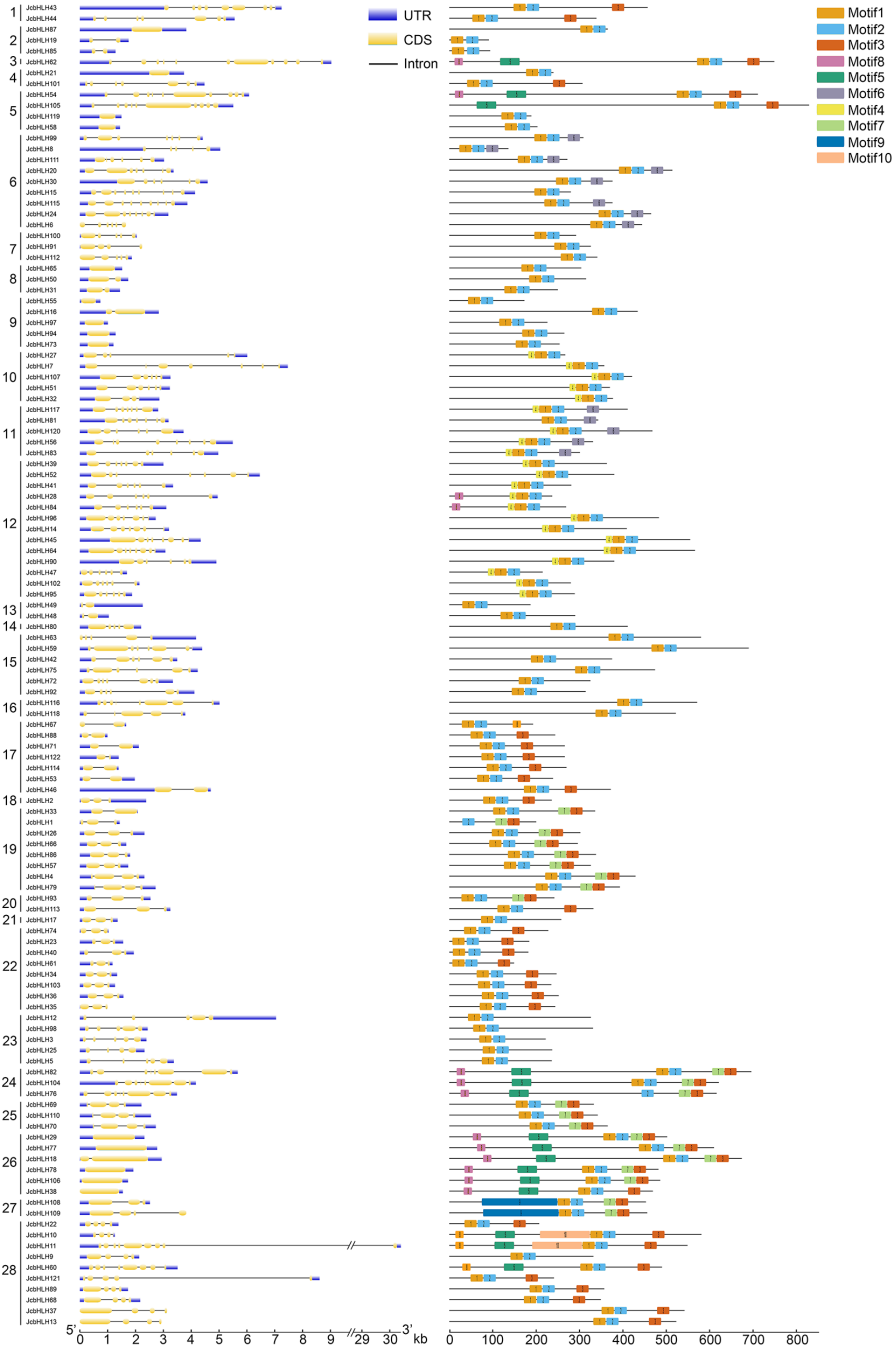
Intron-exon structure (left) and the composition of conserved motifs (right) in the members of the *JcbHLH* family. Yellow boxes and black lines respectively represent the regions of exons and introns, while blue boxes represent the upstream and downstream boundaries of non-coding regions. The length of the box and the line represents the length of the gene. Subfamilies are arranged on the left side of the diagram and are represented by 28 numbers. Different colors represent different motifs which are numbered 1–10. Each ruler is located at the bottom of the diagram.

To elucidate the evolutionary relationship of bHLH genes, 10 conserved motifs were identified through MEME software (Fig. 2). We ascertained that all JcbHLH protein sequences included conserved bHLH motif 1 or 2 except JcbHLH104 which has no motif 1. Most of the bHLH members in groups 1, 3, 17–22 and 24–28 contained motif 3. Genes in the same group have similar motifs, but there are some special motifs in some groups. For example, motif 5, motif 7 and motif 8 were detected in group 24 and 26, motif 4 was detected in group 10, 11 and 12, motif 9 only exists in group 27, and motif 6 was found in groups 6 and 11. Therefore, the *JcbHLHs* clustered in the same group have similar motifs, which helped to clarify the phylogenetic relationship among bHLHs in physic nut.

The exon-intron composition of each JcbHLH was also studied to analyze the evolution of its gene structure (Fig. 2). The numbers of exons in each *JcbHLH* gene vary from 1 to 11, some subfamilies such as 17, 19, 22, 23 and 24 containing 2, 2, 3 ,5 and 8 exons, respectively. Generally speaking, the exons number of genes grouped into the same subfamily was semblable.

### Chromosomal location of JcbHLHs

**Figure 3:**
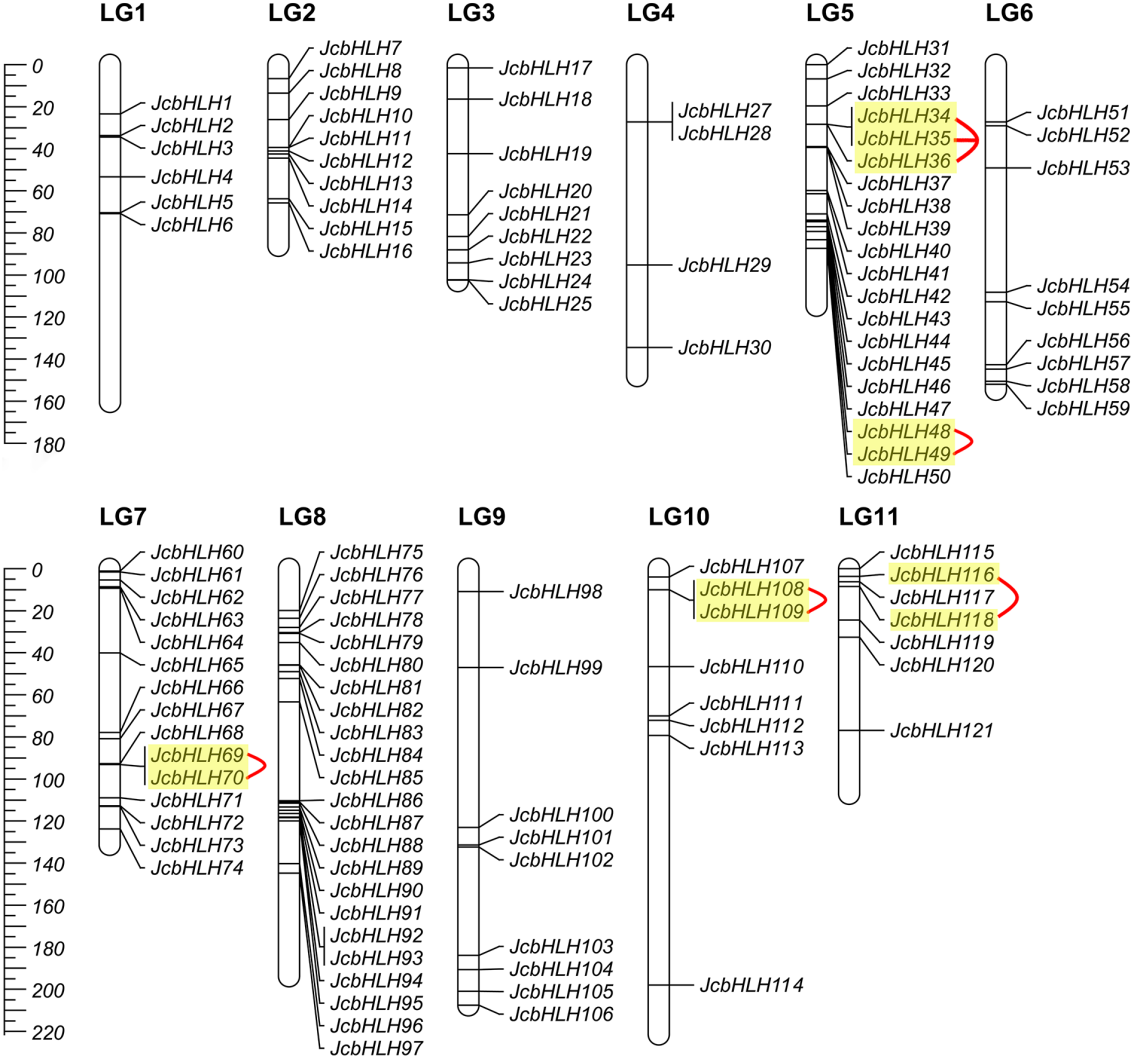
Distribution of *JcbHLH* genes on the physic nut linkage map. 120 JcbHLHs were located on 11 linkage groups (LGs). The scale is measured in centiMorgans (cM). Yellow backgrounds and red curves represent tandem duplicate genes.

Among the 122 *JcbHLH*s, 120 genes were mapped to 11 chromosomes in the physic nut genome except for two genes (*JcbHLH26* and *JcbHLH122*) (Fig. 3). *JcbHLHs* were unevenly distributed on 11 chromosomes. Nine *JcbHLH* genes (7.4%) were on LG3, 6, and 9. Six *JcbHLHs* (5%), 10 *JcbHLHs* (8.2%), 4 *JcbHLHs* (3.3%), 20 *JcbHLHs* (16.4%), 15 *JcbHLHs* (12.3%), 23 *JcbHLHs* (18.9%), 8 *JcbHLHs* (6.6%), and 7 *JcbHLHs* (5.74%) were located on LG1, 2, 4, 5, 7, 8, 10, and 11, respectively.

Furthermore, the result of gene structure analysis manifested that the number of exons and introns in the same subgroup is similar (Fig. 2), indicating a high degree of functional similarity within the same subgroup. In addition, the MEME tool was applied to analyze the motifs of JcbHLH protein sequences. It was found that there were 10 motifs in JcbHLH protein sequences, and the motifs of the same subgroup were similar (Fig. 2). Motif 1 and Motif 2 are highly conserved in all physic nut bHLH proteins except JcbHLH1 which have no Motif 1 (Fig. 2).

### Synteny analysis and duplication of *JcbHLH* genes

**Figure 4:**
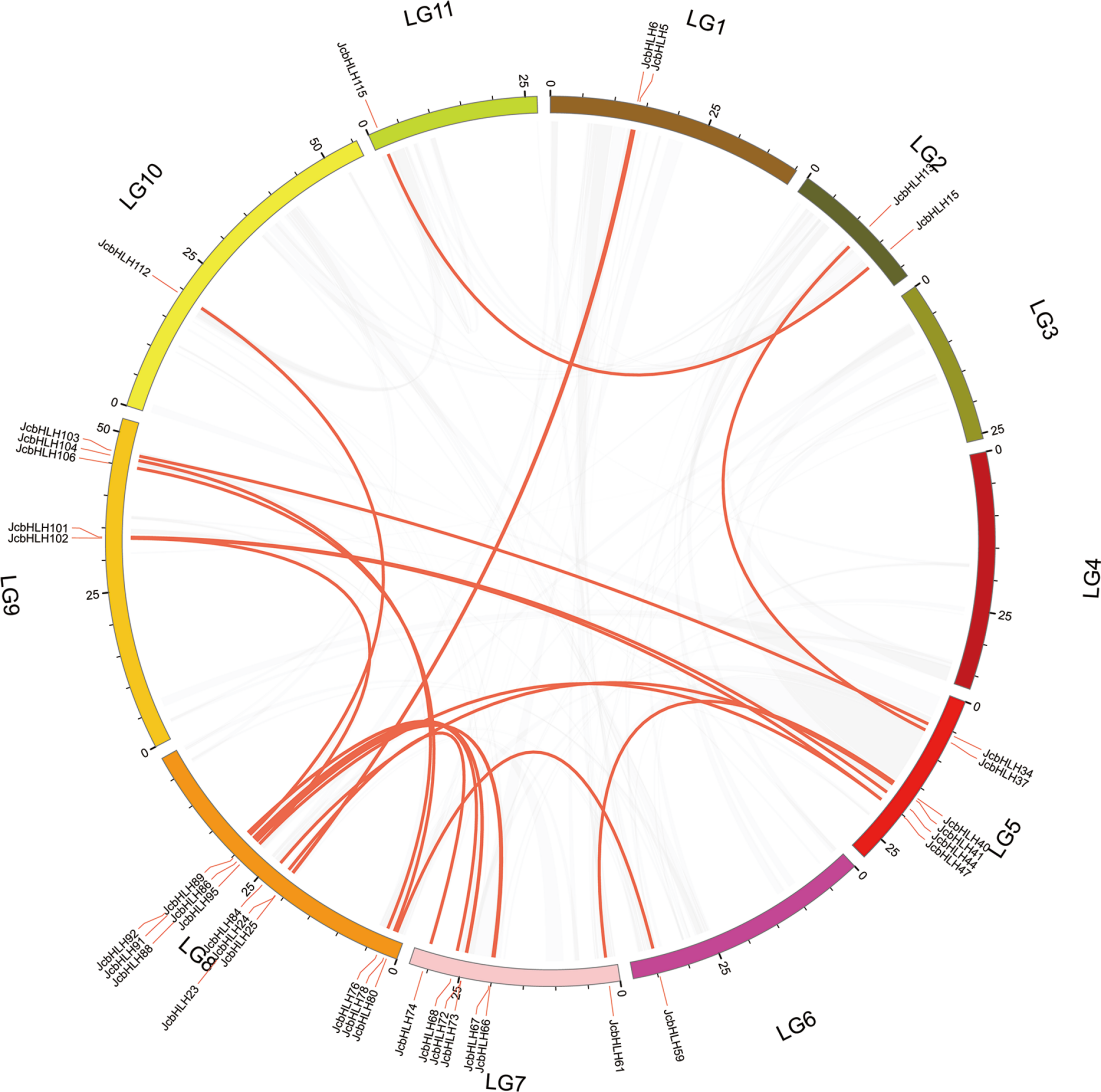
Colinear distribution of the *JcbHLHs*. The gray line in the circle represents the collinearity of the entire genome in *Physic nut* The red line inside the circle represents *JcbHLH* segmental duplications. The red line outside the circle shows the gene location on the chromosome.

The events of segmental replication, tandem replication and transposition are the primary reasons for the expansion and evolution of gene families. Through the MCScanX package, five tandem duplication events containing 11 *JcbHLH* genes were discovered on chromosomes 5, 7, 10 and 11 (Fig. 3). There are two tandem repeat events (*JcbHLH35* and *JcbHLH34*/*JcbHLH36*) related to *JcbHLH35*. We found that all the genes responsible for tandem repetition events come from the same subfamily (Fig. 3). In addition, 21 pairs of segment duplications of the 39 *JcbHLH* genes were detected (Fig. 4). These findings indicated that gene duplication (especially segmental duplication) maybe relate to the amplification of the *JcbHLH* family, and these replication events may be the main driving force of *JcbHLH* evolution.

**Figure 5:**
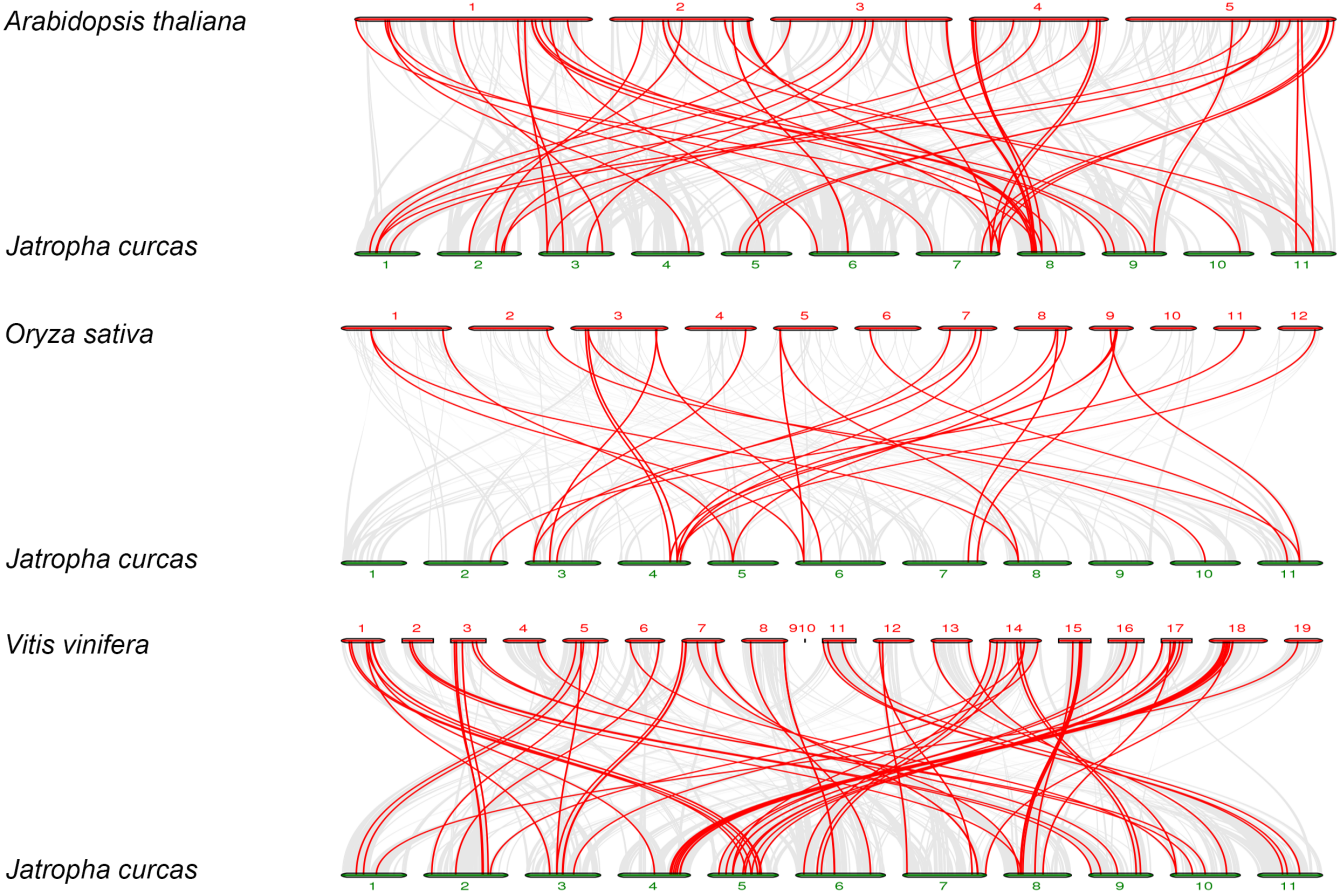
Synteny analyses of the bHLH genes from *Physic nut* with representative plants (*Arabidopsis*, rice and grape). The gray line represents the collinearity of the entire genome. The red line represents the collinearity of the *bHLH* gene family. The numbers represent the chromosomes of each species.

To further explore the phylogenetic mechanisms of *JcbHLHs*, three syntenic maps of physic nut were constructed with three representative plant species (Fig. 5). There were 39, 18 and 64 JcbHLH genes homologous to *Arabidopsis thaliana, Oryza sativa* and *Vitis vinifera*, respectively (Table S4). By comparison, more putative homologous genes were found between physic nut and eudicotyledons than those of monocotyledons. In addition, seven collinear genes (*JcbHLH13*, *JcbHLH39*, *JcbHLH59*, *JcbHLH73*, *JcbHLH80*, *JcbHLH84* and *JcbHLH120*) were found in all four species and three more (*JcbHLH22*, *JcbHLH24* and *JcbHLH66*) in three species (physic nut, *Arabidopsis* and rice), which implies that these genes may play a critical part in the cause of *bHLH* gene family evolution.

### Cis-elements analysis of JcbHLH promoters

Based on previous studies, *bHLH* genes played an important part in many abiotic stresses response (Sun, Wang & Sui, 2018). To analyze the potential cis-elements of the *JcbHLH* gene promoter, the 2 kb promoter region was studied. Our results showed several elements in response to hormones, light, defense, drought, salt, low temperature, development, and so on (Fig. S1). Some *JcbHLH* promoter regions contain MYB binding sites, which may be involved in flavonoid synthesis (Fig. S1). The *JcbHLH* promoter regions containing G-Box and Box-4 elements may respond to light signals. Considering the distribution of *cis*-elements in these gene promoters, we hypothesize that they may involve in regulating the expression of genes related to plant development and stress response.

### Expression pattern of JcbHLHs in different tissues

**Figure 6:**
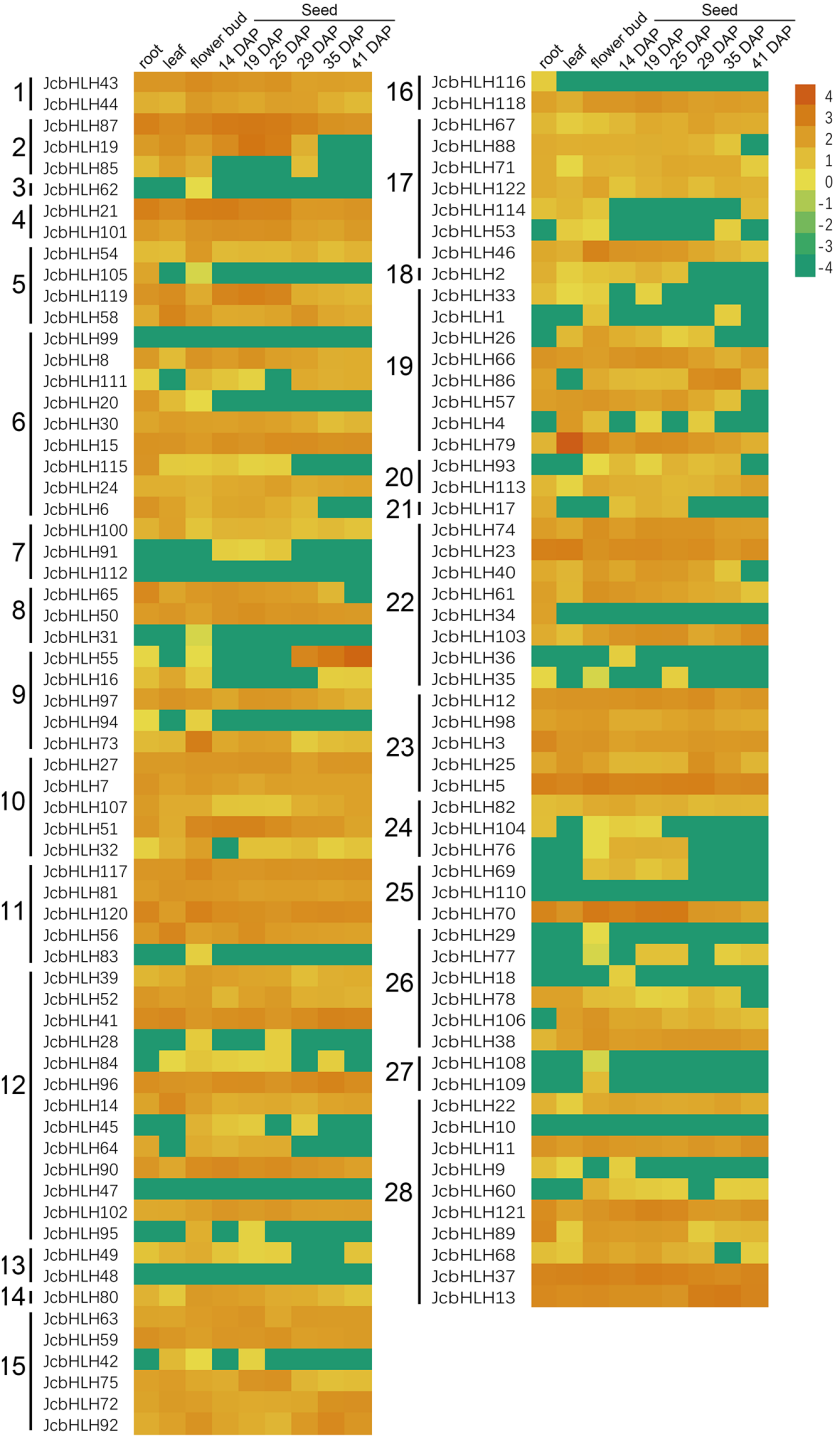
Expression patterns of *JcbHLH* genes family members in different tissues. Heat maps were created based on the value from bHLHs transcriptome data based on lg^(value +0.0001)^ conversion of the FPKMs. The color scale represents signal values. Subfamilies are arranged on the left side of the diagram and are represented by 28 numbers.

To investigate the tissue-specific expression of *JcbHLHs*, the expression profile of *JcbHLHs* was analyzed (Fig. 6). Seven *JcbHLH* genes were expressed in only one tissue, of which six genes (*JcbHLH29*, *JcbHLH31*, *JcbHLH62*, *JcbHLH83*, *JcbHLH108* and *JcbHLH109*) were only expressed in flower buds, while the other one (*JcbHLH116*) was only expressed in roots. In addition, three genes (*JcbHLH18*, *JcbHLH36* and *JcbHLH91*) were only expressed in early developmental seeds, three genes (*JcbHLH85*, *JcbHLH105* and *JcbHLH120*) were expressed in roots and flower buds with no expression in seeds, and six genes (*JcbHLH28*, *JcbHLH95*, *JcbHLH42*, *JcbHLH104*, *JcbHLH76* and *JcbHLH69*) were only expressed in flower bud and early developmental seeds. Among the other 103 *JcbHLHs*, except six genes (*JcbHLH47*, *JcbHLH48*, *JcbHLH10*, *JcbHLH99*, *JcbHLH110 and JcbHLH112*) were not expressed in all samples, other genes had different expression patterns, of which 60 genes were expressed in all samples. It is worth noting that all ten collinear genes mentioned above except *JcbHLH84* were constitutively expressed in all tissues, implying that they play a key role in plant development.

### Expression pattern of JcbHLH genes under salinity and drought treatment

**Figure 7:**
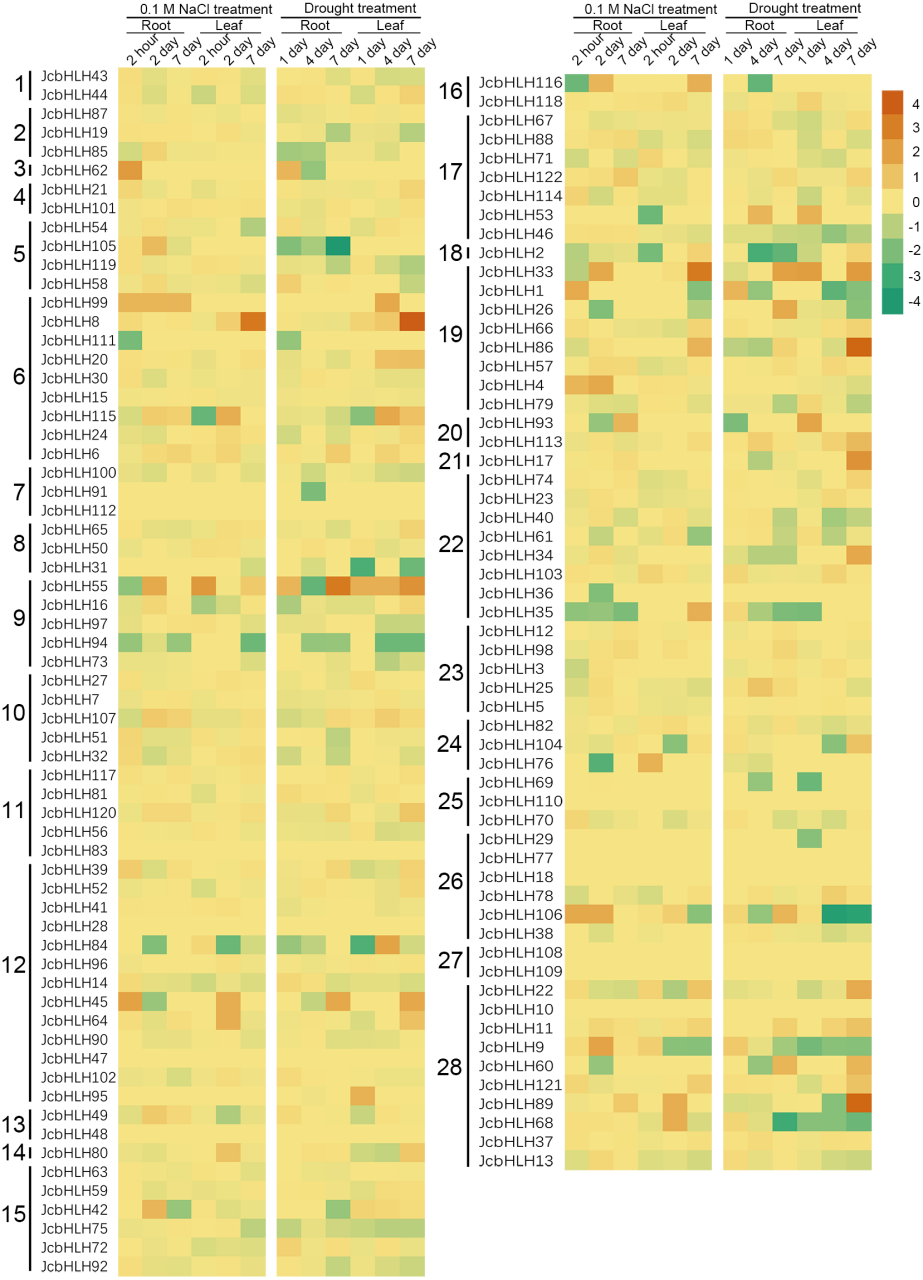
Expression fold changes of *JcbHLH* genes under salinity and drought treatment in leaf and root compared to control. Heat maps were created based on the log_2_^value (treatment/control)^ from bHLHs transcriptome data. The color scale represents signal values. Subfamilies are arranged on the left side of the diagram and are represented by 28 numbers.

For the sake of analyzing the expression pattern of *JcbHLH* genes under salinity and drought treatment in leaves, we analyzed the reported expression profile data (Zhang et al., 2015; Zhang, 2014). Among 122 *JcbHLHs* genes, 96 genes were differentially expressed more than two times at least in one of the time points in roots or leaves under salt (0.1 M NaCl) or drought treatment (Fig. 7). Total 26 genes were not responded under salt and drought treatment, such as *JcbHLH83* in group 11, *JcbHLH18* and *JcbHLH* 77 in group 26 *JcbHLH108* and *JcbHLH109* in group 27, *JcbHLH110* in group 25, *JcbHLH112* in group 7, *JcbHLH10* in group 28, *JcbHLH28* and *JcbHLH47* in group 12, and *JcbHLH48* in group 13. These results manifest that the 26 genes may not involve in salt and drought stress response. As shown in Fig. 7, three genes (*JcbHLH33*, *JcbHLH45* and *JcbHLH55*) were upregulated at least 16 times at most of the time points in roots and leaves under salinity and drought. Moreover, it is worth noting that three genes (*JcbHLH106*, *JcbHLH1* and *JcbHLH62*) and four genes (*JcbHLH8*, *JcbHLH86*, *JcbHLH89* and *JcbHLH115*) were significantly up-regulated in early roots and the middle and later leaves respectively under drought and salt stress. It is suggested that these 10 genes may play a major role in salt and drought stress response. In addition, eight genes (*JcbHLH4*, *JcbHLH9*, *JcbHLH35*, *JcbHLH42*, *JcbHLH68*, *JcbHLH76*, *JcbHLH105* and *JcbHLH116*) and another eight genes (*JcbHLH17*, *JcbHLH26*, *JcbHLH34*, *JcbHLH53*, *JcbHLH60*, *JcbHLH104*, *JcbHLH113* and *JcbHLH121*) were significantly up-regulated only under salinity and drought respectively, implying that they may have some functions in salt and drought response respectively. To facilitate examine the reliability of RNA-seq data, nine collinear genes among the physic nut, *Arabidopsis thaliana*, *Oryza sativa* and *Vitis vinifera* were selected for qRT-PCR verification. This result was in substantial agreement with the expression changes of RNA-seq, suggesting that the data of RNA-seq was exact on the whole.

### Protein interaction network

**Figure 8:**
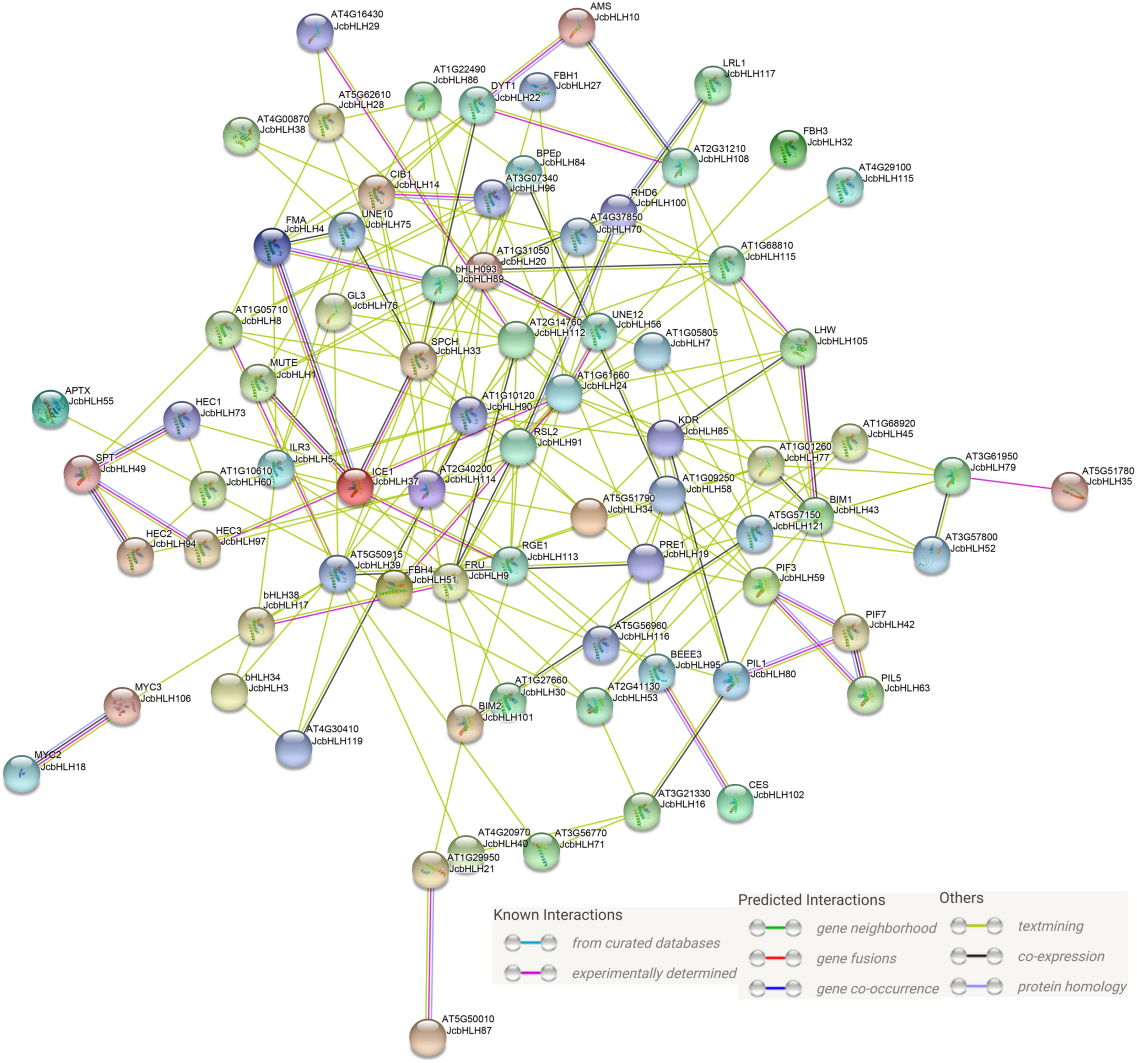
Protein-protein interaction network of JcbHLHs. In the network, the colors of lines and nodes indicate various types and degrees of interaction, respectively. These abbreviations are genes that have been studied in *Arabidopsis thaliana*.

Previous studies showed that bHLH mainly serve a function in the form of homologous dimers or heterologous dimers (Zhu et al., 2015). In our study, protein-protein interaction network prediction of *JcbHLH* was conducted based on *Arabidopsis bHLH* ortholog genes (Fig. 8). The results showed that both AMS (homolog of *JcbHLH10*) and DYT1 (homolog of *JcbHLH 22*) participate in tapetum development regulation. The SPT (homolog of *JcbHLH49*) could interact with HEC (homolog of *JcbHLH73*, *JcbHLH* 94 and *JcbHLH* 97) to regulate carpel fusion and control gynoecium development *via* modulate auxin and cytokinin responses (Schuster, Gaillochet & Lohmann, 2015). The ICE1 (homologous gene of *JcbHLH37*) could interact with FAMA (homologous gene of *JcbHLH4*), SPCH (homologous gene of *JcbHLH33*) and MUTE (homologous gene of *JcbHLH1*) to regulate stomatal development (Qi & Torii, 2018). Moreover, ICE1 is also involved in the response to cold stress (Zhang et al., 2018b). RHD6 (homologous gene of *JcbHLH100*), RSL2 (homologous gene of *JcbHLH91*) and LRL1 (homologous gene of *JcbHLH117*) were involved in the initiation and growth of root hairs respectively (Breuninger et al., 2016; Yi & Keke, 2008). Moreover, the PIF3 (homolog of *JcbHLH59*), PIF7 (homologous gene of *JcbHLH42*), PIL1 (homologous gene of *JcbHLH63*) and PIL1 (homologous gene of *JcbHLH80*), involved in growth and development, were regulated by light. Therefore, JcbHLH genes may be important members of the regulatory networks regulating the development and stress response of physic nut.

## Discussion

As one of the most important transcription factor gene families in plants, bHLHs participated in many essential physiological and developmental processes in various plants, such as *Arabidopsis*, *Zea mays*, tomato and Chinese cabbage (Carretero-Paulet et al., 2010; Song et al., 2014; Sun, Fan & Ling, 2015; Zhang et al., 2018a).

In this study, 122 *JcbHLHs* were detected in physic nut, which is fewer than in *Arabidopsis* (162 genes) (Toledo-Ortiz & Quail, 2003) and rice (167 genes) (Li et al., 2016). One possible explanation is that physic nut experienced fewer recent genome-wide doubling events than *Arabidopsis* and rice, another possible reason is the deletion of *bHLH* gene during the evolution of physic nut (Wu et al., 2015). In addition, five tandem repeat events and 21 pairs of segmental duplications referring to 49 genes were found, indicating that the expansion of the *JcbHLH* gene family could be driven by segmental duplication. Based on the phylogenetic analysis between physic nut and *Arabidopsis*, 122 *JcbHLH* genes and 135 *AtbHLH* genes were classified into 28 groups (Fig. 1). Gene structure and motif analysis revealed that most members of each group shared similar motif and exon/intron organization (Fig. 2), which typical characteristics have also been observed in other plants (Sun, Fan & Ling, 2015; Wang et al., 2019b). It is suggested that each group has similar evolutionary origins and biological functions.

Physic nut is a kind of star tree for biodiesel production, which has a certain tolerance for adversity. Previous studies showed that *bHLH* transcription factors not only participate in plant development, but also stress response (Wang et al., 2019b; Wang et al., 2018).

To better study the *JcbHLH* genes, the analysis of cis-element, protein-protein interaction network and expression profile of the 122 *JcbHLHs* were conducted. The results showed that most *JcbHLH* genes are expressed in roots, leaves, flower buds and developmental seeds (Fig. 6), and their promoters contain a wide range of abiotic stress-responsive elements (ABRE element, TC-rich element, MBS element, DRE element and LTR element) (Fig. S1), which indicates that these *JcbHLH* genes may play critical roles in physic nut development and stress response. Especially, the *JcbHLH33*, *JcbHLH55* and *JcbHLH45* were most highly upregulated under salt and drought stress (Fig. 7), indicating that these genes may function in both stresses.

Based on the phylogenetic analysis of the *bHLH* gene family of physic nut and *Arabidopsis* (Fig. 1), we further analyzed the function of *JcbHLH* genes according to *AtbHLH* function. In the subfamily 21 all four *AtbHLH* genes (*At3G56970*, *At3G56980*, *At2G41240* and *At5G04150*) are play important roles in regulating iron homeostasis under Fe deficiency in *Arabidopsis* (Li et al., 2016; Liang et al., 2017), which means the same subfamily member *JcbHLH17* may also perform similar functions in physic nut. The significantly upregulated of *JcbHLH17* gene in the leaves of physic nut seedings treated with drought for seven days (Fig. 7) further inferred the similar mechanism response to drought stress based on regulating iron homeostasis in physic nut. In the subfamily *19*, *At3G06120*, *At5G53210* and *At3G24140* have been shown to be closely related to stomatal development (Liu, Ohashi-Ito & Bergmann, 2009; Pillitteri et al., 2007). As expected, *JcbHLH1*, *JcbHLH4* and *JcbHLH33* (subfamily 19) were significantly up-regulated in at least two samples treated with salt and drought stress (Fig. 7), so it is speculated that *JcbHLH1*, *JcbHLH4* and *JcbHLH33* response to salt and drought stress may through the regulation of stomatal development. On the other hand, the three genes show different expression patterns in physic nut (Fig. 7), indicating that their functions have differentiated, or they may perform the same function in different ways. In *Arabidopsis*, *AtbHLH112* regulates the pathways of proline biosynthesis and ROS scavenging to enhance their stress tolerance (Liu et al., 2015). The *JcbHLH99* (the putative homologous gene of *AtbHLH112*) significantly up-regulated in roots at different stages under salt stress, may play roles in the same way.

Protein interaction network analysis showed that many JcbHLH proteins may play important roles in regulating physic nut gene expression and metabolic processes by forming complexes (Fig. 8), which is consistent with previous studies (Zhu et al., 2015). For instance, the putative *AtMYC3* homolog *JcbHLH106* was also significantly upregulated in roots under salt and drought stress (Fig. 7), which may, like MYC3 in *Arabidopsis*, activate the JA-responses with MYC2 and MYC4, thereby enhancing physical nut tolerance (Zhang et al., 2020a).

Finally, ten genes were screened by genomic collinearity analysis among physic nut, *Arabidopsis thaliana*, and *Oryza sativa.* The expression profile manifested that all collinearity genes were highly expressed in different tissues except *JcbHLH84*, which further indicates the critical role in the growth of physic nut.

All in all, these results provide a good foundation for further study of bHLH gene family function in physic nut.

## Conclusions

In this study, 122 *JcbHLH* genes were detected in the genome of physic nut, and further classified into 28 groups. Through the analysis of chromosome location, gene structure, phylogeny and collinearity, the relationships among family members were elucidated. By analyzing the expression profiles of these genes in various tissues and stress treatment, the expression patterns of all members were studied in physic nut. Many *JcbHLH* genes have been proved to be closely related to the development and stress response of the physic nut. In addition, a complete protein-protein interaction network of JcbHLHs was predicted and some important genes in growth and development were excavated. These discoveries lay a theoretical foundation for further studies on the biological function of *JcbHLHs*, and have important significance in the molecular breeding of physic nut and other plants.

## Supplemental Information

10.7717/peerj.13786/supp-1 Supplemental Information 1 Cis-element analysis of promoter regions of *JcbHLH* genes Different cis-elements were represented by different colored boxes.

10.7717/peerj.13786/supp-2 Supplemental Information 2 Primers used in this study

10.7717/peerj.13786/supp-3 Supplemental Information 3 The bHLH protein sequences of *Arabidopsis* and Rice

10.7717/peerj.13786/supp-4 Supplemental Information 4 Accession members and characteristics of 121 JcbHLH genes in physic nut

10.7717/peerj.13786/supp-5 Supplemental Information 5 One-to-one orthologous relationships between *Jatropha curcas* and other plants

## Additional Information and Declarations

### Data Availability

The following information was supplied regarding data availability: The data is available at NCBI SRA: PRJNA257901, PRJNA244896 and SRR2039597 and NCBI Genome: AFEW00000000.
