# Supplementary figures and images for: Genome-wide characterization and expression analysis of bHLH gene family in physic nut (Jatropha curcas L.)
Source: PeerJ. 2022 Aug 9;10:e13786. doi: 10.7717/peerj.13786 (PMC9373979; doi:10.7717/peerj.13786)

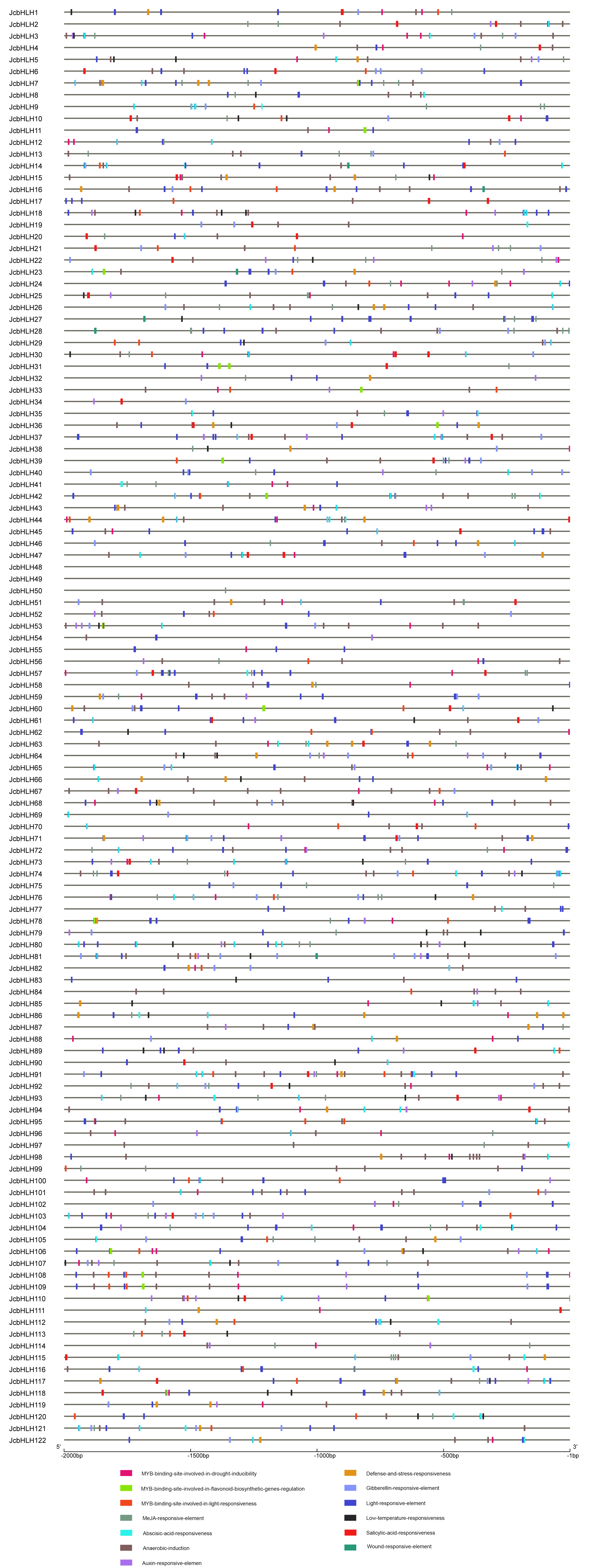

Supplement: Supplemental Information 1 — Different cis-elements were represented by different colored boxes. [file peerj-10-13786-s001.png]
