# Supplementary material for: Genome-wide characterization and expression analysis of bHLH gene family in physic nut (Jatropha curcas L.)
Source: PeerJ. 2022 Aug 9;10:e13786. doi: 10.7717/peerj.13786 (PMC9373979; doi:10.7717/peerj.13786)
Supplement: Supplemental Information 2 [file peerj-10-13786-s002.docx]

**Table S3 Primers used in this study.**

| Primer Name | Forward Primer sequences (5’>3’) | Reverse Primer sequences (5’>3’) |
| --- | --- | --- |
| JcbHLH13 | ACCCTTTGACTCCTACACCT | TCCCTTACCCTAACTTCCA |
| JcbHLH22 | TGGAATAATGGAAGAAGTTCAGGTG | TGGTGTCAGTGAGTTCAAGGC |
| JcbHLH24 | ATAACCGAACAATCAACTACTCCTGC | TGCTGCTTTGGCAATGAGG |
| JcbHLH39 | CAAGAATCTATCTGGCTTTATGGAG | CAGCGTAGTAAAATCTAACATCAGG |
| JcbHLH59 | AATGAATGCTGGTGGGGTGG | GCATTGTTCGTGAGTTGACATTTTG |
| JcbHLH66 | AAGTTCCGATAACCAGTTCAGGC | GATGCTTGGTCTCCCCTTTGA |
| JcbHLH73 | GAATAAGCGAAAGGATAAGG | AAGCCAGCCATTGGTCTA |
| JcbHLH80 | ACAAACACCTCCAGATGAGCAAT | GAGCATACTGAAGCGGAAGCA |
| JcbHLH120 | GCCACCACCATCATTTACT | ACGGAGGCAGCATCTTTA |
