# Supplementary material for: Genome-wide characterization and expression analysis of bHLH gene family in physic nut (Jatropha curcas L.)
Source: PeerJ. 2022 Aug 9;10:e13786. doi: 10.7717/peerj.13786 (PMC9373979; doi:10.7717/peerj.13786)
Supplement: Supplemental Information 4 [file peerj-10-13786-s004.docx]

**Table S1 Accession members and characteristics of 121 JcbHLH genes in physic nut.**

| **Genes** | **Gene ID** | **Protein length(aa)** | **pI** | **MW**  **(kDa)** | **Loaction** |
| --- | --- | --- | --- | --- | --- |
| *JcbHLH1* | LOC105631917 | 200 | 8.35 | 22.4 | LG1 |
| *JcbHLH2* | LOC105632090 | 236 | 8.27 | 27.4 | LG1 |
| *JcbHLH3* | LOC105632116 | 222 | 5.74 | 25.1 | LG1 |
| *JcbHLH4* | LOC105640465 | 281 | 5.73 | 30.8 | LG1 |
| *JcbHLH4* | LOC105633186 | 429 | 5.57 | 48.9 | LG1 |
| *JcbHLH5* | LOC105632874 | 236 | 7.7 | 25.7 | LG1 |
| *JcbHLH6* | LOC105632848 | 444 | 6.16 | 49.3 | LG1 |
| *JcbHLH7* | LOC105629297 | 357 | 8.62 | 38.3 | LG2 |
| *JcbHLH8* | LOC105628270 | 136 | 9.05 | 15.1 | LG2 |
| *JcbHLH9* | LOC105628307 | 332 | 5.12 | 36.9 | LG2 |
| *JcbHLH10* | LOC105641820 | 581 | 5.31 | 66.1 | LG2 |
| *JcbHLH11* | LOC105641818 | 549 | 5.36 | 62.0 | LG2 |
| *JcbHLH12* | LOC105643782 | 326 | 6.55 | 36.0 | LG2 |
| *JcbHLH13* | LOC105630202 | 523 | 5.34 | 57.2 | LG2 |
| *JcbHLH14* | LOC105630155 | 409 | 6.91 | 45.5 | LG2 |
| *JcbHLH15* | LOC105635021 | 280 | 6.35 | 30.8 | LG2 |
| *JcbHLH16* | LOC105631694 | 434 | 6.33 | 48.4 | LG2 |
| *JcbHLH17* | LOC105634421 | 258 | 7.1 | 29.4 | LG3 |
| *JcbHLH18* | LOC105637396 | 674 | 5.42 | 74.0 | LG3 |
| *JcbHLH19* | LOC105642800 | 91 | 7.94 | 10.5 | LG3 |
| *JcbHLH20* | LOC105634210 | 514 | 6.02 | 56.6 | LG3 |
| *JcbHLH21* | LOC105633966 | 240 | 6.08 | 26.9 | LG3 |
| *JcbHLH22* | LOC105650123 | 207 | 5.48 | 23.6 | LG3 |
| *JcbHLH23* | LOC105649551 | 184 | 8.69 | 21.0 | LG3 |
| *JcbHLH24* | LOC105649417 | 465 | 5.39 | 50.8 | LG3 |
| *JcbHLH25* | LOC105649400 | 237 | 5.65 | 26.0 | LG3 |
| *JcbHLH26* | LOC105638657 | 302 | 7.14 | 34.3 | - |
| *JcbHLH27* | LOC105636736 | 267 | 5.39 | 29.0 | LG4 |
| *JcbHLH28* | LOC105636735 | 237 | 6.01 | 25.9 | LG4 |
| *JcbHLH29* | LOC105645115 | 502 | 5.76 | 55.7 | LG4 |
| *JcbHLH30* | LOC105633836 | 376 | 6.75 | 41.1 | LG4 |
| *JcbHLH31* | LOC105646251 | 250 | 4.93 | 28.4 | LG5 |
| *JcbHLH32* | LOC105650469 | 377 | 7.2 | 42.3 | LG5 |
| *JcbHLH33* | LOC105648363 | 336 | 5.16 | 36.9 | LG5 |
| *JcbHLH34* | LOC105648860 | 247 | 5.35 | 27.5 | LG5 |
| *JcbHLH35* | LOC105648861 | 244 | 6.33 | 28.0 | LG5 |
| *JcbHLH36* | LOC105648547 | 252 | 9.05 | 28.4 | LG5 |
| *JcbHLH37* | LOC105648729 | 542 | 5.68 | 59.1 | LG5 |
| *JcbHLH38* | LOC105648893 | 469 | 8 | 52.7 | LG5 |
| *JcbHLH39* | LOC105648738 | 363 | 7.05 | 40.6 | LG5 |
| *JcbHLH40* | LOC105640400 | 182 | 9.32 | 21.0 | LG5 |
| *JcbHLH42* | LOC105640588 | 375 | 7.78 | 41.3 | LG5 |
| *JcbHLH43* | LOC105640676 | 457 | 8.15 | 50.0 | LG5 |
| *JcbHLH44* | LOC105640688 | 339 | 7.08 | 37.5 | LG5 |
| *JcbHLH45* | LOC105640700 | 555 | 5.8 | 60.0 | LG5 |
| *JcbHLH46* | LOC105641974 | 372 | 6.05 | 41.4 | LG5 |
| *JcbHLH47* | LOC105642084 | 215 | 6.08 | 24.4 | LG5 |
| *JcbHLH48* | LOC105636978 | 290 | 6.79 | 32.0 | LG5 |
| *JcbHLH49* | LOC105636977 | 187 | 9.32 | 21.0 | LG5 |
| *JcbHLH50* | LOC105636938 | 315 | 4.85 | 35.9 | LG5 |
| *JcbHLH51* | LOC105634739 | 370 | 8.89 | 40.9 | LG6 |
| *JcbHLH52* | LOC105634733 | 380 | 5.74 | 42.1 | LG6 |
| *JcbHLH53* | *LOC105643894* | *239* | *8.64* | *26.9* | *LG6* |
| *JcbHLH54* | LOC105646707 | 711 | 5.44 | 79.3 | LG6 |
| *JcbHLH55* | LOC105648118 | 173 | 7.87 | 19.6 | LG6 |
| *JcbHLH56* | LOC105647690 | 331 | 5.63 | 34.8 | LG6 |
| *JcbHLH57* | LOC105647578 | 326 | 5.39 | 37.0 | LG6 |
| *JcbHLH58* | LOC105647433 | 203 | 11.16 | 23.1 | LG6 |
| *JcbHLH59* | LOC105647368 | 690 | 6.04 | 74.1 | LG6 |
| *JcbHLH60* | LOC105644598 | 490 | 9.03 | 54.6 | LG7 |
| *JcbHLH61* | LOC105644965 | 149 | 7.77 | 16.7 | LG7 |
| *JcbHLH62* | LOC105644561 | 749 | 6.05 | 83.1 | LG7 |
| *JcbHLH63* | LOC105644435 | 580 | 6.26 | 63.3 | LG7 |
| *JcbHLH64* | LOC105644426 | 566 | 6.17 | 62.0 | LG7 |
| *JcbHLH65* | LOC105644026 | 304 | 8.58 | 33.9 | LG7 |
| *JcbHLH66* | LOC105641739 | 296 | 6.01 | 33.8 | LG7 |
| *JcbHLH67* | LOC105641744 | 193 | 9.76 | 21.8 | LG7 |
| *JcbHLH68* | LOC105639930 | 349 | 4.8 | 39.4 | LG7 |
| *JcbHLH69* | LOC105639913 | 333 | 9.15 | 37.0 | LG7 |
| *JcbHLH70* | LOC105640003 | 365 | 5.93 | 41.0 | LG7 |
| *JcbHLH71* | LOC105639576 | 266 | 6.67 | 29.4 | LG7 |
| *JcbHLH72* | LOC105639723 | 325 | 5.65 | 34.6 | LG7 |
| *JcbHLH73* | LOC105639713 | 254 | 9.49 | 28.3 | LG7 |
| *JcbHLH74* | LOC105646925 | 228 | 7.73 | 25.9 | LG7 |
| *JcbHLH75* | LOC105643171 | 474 | 7.99 | 51.1 | LG8 |
| *JcbHLH76* | LOC105642985 | 616 | 5.12 | 69.8 | LG8 |
| *JcbHLH77* | LOC105642836 | 610 | 6.26 | 67.8 | LG8 |
| *JcbHLH78* | LOC105642791 | 482 | 5.31 | 54.0 | LG8 |
| *JcbHLH79* | LOC105642767 | 393 | 6.06 | 45.0 | LG8 |
| *JcbHLH80* | LOC105642714 | 411 | 8.74 | 46.0 | LG8 |
| *JcbHLH81* | LOC105650022 | 343 | 5.37 | 38.8 | LG8 |
| *JcbHLH82* | LOC105650007 | 696 | 5.33 | 77.6 | LG8 |
| *JcbHLH83* | LOC105649906 | 301 | 5.74 | 31.9 | LG8 |
| *JcbHLH84* | LOC105649806 | 269 | 6.93 | 29.5 | LG8 |
| *JcbHLH85* | LOC105630410 | 94 | 7.94 | 10.6 | LG8 |
| *JcbHLH86* | LOC105630661 | 338 | 5.9 | 37.5 | LG8 |
| *JcbHLH87* | LOC105630681 | 365 | 4.76 | 40.6 | LG8 |
| *JcbHLH88* | LOC105630700 | 244 | 9.16 | 27.1 | LG8 |
| *JcbHLH58* | LOC105647433 | 203 | 11.16 | 23.1 | LG6 |
| *JcbHLH59* | LOC105647368 | 690 | 6.04 | 74.1 | LG6 |
| *JcbHLH60* | LOC105644598 | 490 | 9.03 | 54.6 | LG7 |
| *JcbHLH61* | LOC105644965 | 149 | 7.77 | 16.7 | LG7 |
| *JcbHLH62* | LOC105644561 | 749 | 6.05 | 83.1 | LG7 |
| *JcbHLH63* | LOC105644435 | 580 | 6.26 | 63.3 | LG7 |
| *JcbHLH64* | LOC105644426 | 566 | 6.17 | 62.0 | LG7 |
| *JcbHLH65* | LOC105644026 | 304 | 8.58 | 33.9 | LG7 |
| *JcbHLH66* | LOC105641739 | 296 | 6.01 | 33.8 | LG7 |
| *JcbHLH67* | LOC105641744 | 193 | 9.76 | 21.8 | LG7 |
| *JcbHLH68* | LOC105639930 | 349 | 4.8 | 39.4 | LG7 |
| *JcbHLH69* | LOC105639913 | 333 | 9.15 | 37.0 | LG7 |
| *JcbHLH70* | LOC105640003 | 365 | 5.93 | 41.0 | LG7 |
| *JcbHLH71* | LOC105639576 | 266 | 6.67 | 29.4 | LG7 |
| *JcbHLH72* | LOC105639723 | 325 | 5.65 | 34.6 | LG7 |
| *JcbHLH73* | LOC105639713 | 254 | 9.49 | 28.3 | LG7 |
| *JcbHLH74* | LOC105646925 | 228 | 7.73 | 25.9 | LG7 |
| *JcbHLH75* | LOC105643171 | 474 | 7.99 | 51.1 | LG8 |
| *JcbHLH76* | LOC105642985 | 616 | 5.12 | 69.8 | LG8 |
| *JcbHLH77* | LOC105642836 | 610 | 6.26 | 67.8 | LG8 |
| *JcbHLH78* | LOC105642791 | 482 | 5.31 | 54.0 | LG8 |
| *JcbHLH79* | LOC105642767 | 393 | 6.06 | 45.0 | LG8 |
| *JcbHLH80* | LOC105642714 | 411 | 8.74 | 46.0 | LG8 |
| *JcbHLH81* | LOC105650022 | 343 | 5.37 | 38.8 | LG8 |
| *JcbHLH82* | LOC105650007 | 696 | 5.33 | 77.6 | LG8 |
| *JcbHLH83* | LOC105649906 | 301 | 5.74 | 31.9 | LG8 |
| *JcbHLH84* | LOC105649806 | 269 | 6.93 | 29.5 | LG8 |
| *JcbHLH85* | LOC105630410 | 94 | 7.94 | 10.6 | LG8 |
| *JcbHLH86* | LOC105630661 | 338 | 5.9 | 37.5 | LG8 |
| *JcbHLH87* | LOC105630681 | 365 | 4.76 | 40.6 | LG8 |
| *JcbHLH88* | LOC105630700 | 244 | 9.16 | 27.1 | LG8 |
| *JcbHLH89* | LOC105630754 | 357 | 4.62 | 40.8 | LG8 |
| *JcbHLH90* | LOC105630806 | 380 | 6.06 | 41.5 | LG8 |
| *JcbHLH91* | LOC105631009 | 326 | 5.04 | 36.4 | LG8 |
| *JcbHLH92* | LOC105630933 | 314 | 5.92 | 34.0 | LG8 |
| *JcbHLH93* | LOC105630934 | 242 | 7.74 | 26.9 | LG8 |
| *JcbHLH94* | LOC105630947 | 265 | 8.49 | 29.5 | LG8 |
| *JcbHLH95* | LOC105650687 | 289 | 5.95 | 33.1 | LG8 |
| *JcbHLH96* | LOC105639096 | 483 | 5.69 | 52.9 | LG8 |
| *JcbHLH97* | LOC105641859 | 226 | 5.22 | 25.9 | LG8 |
| *JcbHLH98* | LOC105629018 | 331 | 5.56 | 36.8 | LG9 |
| *JcbHLH99* | LOC105634633 | 309 | 5.78 | 34.5 | LG9 |
| *JcbHLH100* | LOC105638607 | 292 | 6.18 | 32.5 | LG9 |
| *JcbHLH101* | LOC105646782 | 307 | 5.33 | 34.4 | LG9 |
| *JcbHLH102* | LOC105638735 | 280 | 8.15 | 31.7 | LG9 |
| *JcbHLH103* | LOC105645913 | 235 | 6 | 26.5 | LG9 |
| *JcbHLH104* | LOC105645803 | 621 | 5.62 | 69.4 | LG9 |
| *JcbHLH105* | LOC105645618 | 829 | 4.73 | 90.3 | LG9 |
| *JcbHLH106* | LOC105645585 | 486 | 8.06 | 54.7 | LG9 |
| *JcbHLH107* | LOC105628675 | 421 | 8.07 | 46.8 | LG10 |
| *JcbHLH108* | LOC105628859 | 453 | 5.2 | 50.5 | LG10 |
| *JcbHLH109* | LOC105628860 | 456 | 5.15 | 50.4 | LG10 |
| *JcbHLH110* | LOC105635995 | 342 | 6.68 | 38.4 | LG10 |
| *JcbHLH111* | LOC105650843 | 272 | 8.43 | 29.1 | LG10 |
| *JcbHLH112* | LOC105650773 | 341 | 5.12 | 37.6 | LG10 |
| *JcbHLH113* | LOC105639494 | 332 | 5.89 | 36.9 | LG10 |
| *JcbHLH114* | LOC105645984 | 270 | 8.88 | 30.1 | LG10 |
| *JcbHLH115* | LOC105637750 | 376 | 6.86 | 41.4 | LG11 |
| *JcbHLH116* | LOC105638346 | 571 | 6.64 | 64.7 | LG11 |
| *JcbHLH117* | LOC105638024 | 411 | 5.58 | 44.0 | LG11 |
| *JcbHLH118* | LOC105638345 | 522 | 8.55 | 59.5 | LG11 |
| *JcbHLH119* | LOC105633595 | 189 | 11.09 | 21.7 | LG11 |
| *JcbHLH120* | LOC105633497 | 468 | 6.15 | 49.3 | LG11 |
| *JcbHLH121* | LOC105629629 | 241 | 5.27 | 27.5 | LG11 |
| *JcbHLH122* | LOC105636066 | 266 | 9.04 | 29.3 | - |
