# Supplementary material for: Genome-wide characterization and expression analysis of bHLH gene family in physic nut (Jatropha curcas L.)
Source: PeerJ. 2022 Aug 9;10:e13786. doi: 10.7717/peerj.13786 (PMC9373979; doi:10.7717/peerj.13786)
Supplement: Supplemental Information 5 [file peerj-10-13786-s005.docx]

**Table S2 One-to-one orthologous relationships between Jatropha curcas and other plants.**

| ***JcbHLH*** | ***AtbHLH*** |  | ***JcbHLH*** | ***OsbHLH*** |  | ***JcbHLH*** | ***VvbHLH*** |
| --- | --- | --- | --- | --- | --- | --- | --- |
| *JcbHLH1* | *AT3G06120* |  | *JcbHLH13* | *BGIOSGA035358* |  | *JcbHLH1* | *VIT_05s0020g02700* |
| *JcbHLH2* | *AT5G43650* |  | *JcbHLH22* | *BGIOSGA025912* |  | *JcbHLH3* | *VIT_05s0049g00460* |
| *JcbHLH3* | *AT3G23210* |  | *JcbHLH24* | *BGIOSGA017200* |  | *JcbHLH5* | *VIT_19s0014g05100* |
| *JcbHLH3* | *AT4G14410* |  | *JcbHLH37* | *BGIOSGA000206* |  | *JcbHLH7* | *VIT_05s0124g00240* |
| *JcbHLH5* | *AT5G54680* |  | *JcbHLH39* | *BGIOSGA026577* |  | *JcbHLH9* | *VIT_06s0061g00720* |
| *JcbHLH9* | *AT2G28160* |  | *JcbHLH56* | *BGIOSGA013729* |  | *JcbHLH11* | *VIT_03s0038g02540* |
| *JcbHLH11* | *AT2G16910* |  | *JcbHLH59* | *BGIOSGA001870* |  | *JcbHLH12* | *VIT_03s0038g01790* |
| *JcbHLH13* | *AT3G26744* |  | *JcbHLH59* | *BGIOSGA019157* |  | *JcbHLH13* | *VIT_14s0068g01200* |
| *JcbHLH14* | *AT4G34530* |  | *JcbHLH66* | *BGIOSGA026779* |  | *JcbHLH14* | *VIT_03s0038g04760* |
| *JcbHLH19* | *AT3G28857* |  | *JcbHLH73* | *BGIOSGA030896* |  | *JcbHLH16* | *VIT_09s0002g02700* |
| *JcbHLH21* | *AT1G29950* |  | *JcbHLH80* | *BGIOSGA001870* |  | *JcbHLH19* | *VIT_14s0108g00420* |
| *JcbHLH22* | *AT4G21330* |  | *JcbHLH80* | *BGIOSGA019157* |  | *JcbHLH33* | *VIT_16s0050g02500* |
| *JcbHLH24* | *AT1G61660* |  | *JcbHLH83* | *BGIOSGA025266* |  | *JcbHLH36* | *VIT_16s0022g02270* |
| *JcbHLH36* | *AT5G51780* |  | *JcbHLH84* | *BGIOSGA009622* |  | *JcbHLH37* | *VIT_14s0068g01200* |
| *JcbHLH39* | *AT5G50915* |  | *JcbHLH86* | *BGIOSGA012015* |  | *JcbHLH37* | *VIT_17s0000g00330* |
| *JcbHLH46* | *AT1G68810* |  | *JcbHLH86* | *BGIOSGA030936* |  | *JcbHLH39* | *VIT_17s0000g00430* |
| *JcbHLH51* | *AT2G42280* |  | *JcbHLH91* | *BGIOSGA035917* |  | *JcbHLH40* | *VIT_17s0000g08150* |
| *JcbHLH52* | *AT2G42300* |  | *JcbHLH91* | *BGIOSGA011251* |  | *JcbHLH41* | *VIT_01s0026g01140* |
| *JcbHLH59* | *AT1G09530* |  | *JcbHLH91* | *BGIOSGA023988* |  | *JcbHLH41* | *VIT_17s0000g05370* |
| *JcbHLH60* | *AT1G10610* |  | *JcbHLH94* | *BGIOSGA026828* |  | *JcbHLH42* | *VIT_17s0000g06930* |
| *JcbHLH66* | *AT5G65320* |  | *JcbHLH94* | *BGIOSGA030896* |  | *JcbHLH43* | *VIT_17s0000g04790* |
| *JcbHLH68* | *AT5G65640* |  | *JcbHLH112* | *BGIOSGA011251* |  | *JcbHLH44* | *VIT_01s0010g00540* |
| *JcbHLH70* | *AT2G22750* |  | *JcbHLH117* | *BGIOSGA021743* |  | *JcbHLH45* | *VIT_01s0010g00740* |
| *JcbHLH70* | *AT2G22760* |  | *JcbHLH120* | *BGIOSGA009202* |  | *JcbHLH46* | *VIT_01s0011g02940* |
| *JcbHLH70* | *AT4G37850* |  | *JcbHLH120* | *BGIOSGA029759* |  | *JcbHLH47* | *VIT_01s0011g03720* |
| *JcbHLH72* | *AT4G36930* |  |  |  |  | *JcbHLH51* | *VIT_08s0007g07810* |
| *JcbHLH73* | *AT3G50330* |  |  |  |  | *JcbHLH52* | *VIT_08s0007g07870* |
| *JcbHLH73* | *AT5G67060* |  |  |  |  | *JcbHLH55* | *VIT_08s0058g00110* |
| *JcbHLH76* | *AT1G63650* |  |  |  |  | *JcbHLH56* | *VIT_14s0030g02230* |
| *JcbHLH76* | *AT4G00480* |  |  |  |  | *JcbHLH57* | *VIT_14s0128g00110* |
| *JcbHLH77* | *AT1G01260* |  |  |  |  | *JcbHLH59* | *VIT_14s0060g00260* |
| *JcbHLH77* | *AT2G46510* |  |  |  |  | *JcbHLH68* | *VIT_18s0001g08040* |
| *JcbHLH78* | *AT4G00870* |  |  |  |  | *JcbHLH71* | *VIT_12s0028g02350* |
| *JcbHLH79* | *AT2G46810* |  |  |  |  | *JcbHLH72* | *VIT_07s0031g00450* |
| *JcbHLH79* | *AT3G61950* |  |  |  |  | *JcbHLH73* | *VIT_07s0031g00550* |
| *JcbHLH79* | *AT4G01460* |  |  |  |  | *JcbHLH74* | *VIT_12s0028g03550* |
| *JcbHLH80* | *AT2G46970* |  |  |  |  | *JcbHLH75* | *VIT_15s0021g02690* |
| *JcbHLH80* | *AT3G62090* |  |  |  |  | *JcbHLH77* | *VIT_15s0046g00320* |
| *JcbHLH84* | *AT1G59640* |  |  |  |  | *JcbHLH78* | *VIT_15s0048g02820* |
| *JcbHLH90* | *AT1G10120* |  |  |  |  | *JcbHLH78* | *VIT_02s0025g02610* |
| *JcbHLH95* | *AT1G73830* |  |  |  |  | *JcbHLH79* | *VIT_15s0048g02510* |
| *JcbHLH100* | *AT1G66470* |  |  |  |  | *JcbHLH80* | *VIT_07s0005g02510* |
| *JcbHLH101* | *AT5G38860* |  |  |  |  | *JcbHLH81* | *VIT_07s0104g00250* |
| *JcbHLH104* | *AT1G63650* |  |  |  |  | *JcbHLH82* | *VIT_07s0104g00090* |
| *JcbHLH105* | *AT1G64625* |  |  |  |  | *JcbHLH83* | *VIT_05s0020g04620* |
| *JcbHLH114* | *AT2G40200* |  |  |  |  | *JcbHLH83* | *VIT_07s0141g00220* |
| *JcbHLH116* | *AT5G56960* |  |  |  |  | *JcbHLH84* | *VIT_01s0026g01140* |
| *JcbHLH120* | *AT2G24260* |  |  |  |  | *JcbHLH85* | *VIT_01s0026g02030* |
| *JcbHLH120* | *AT5G58010* |  |  |  |  | *JcbHLH86* | *VIT_18s0001g06650* |
|  |  |  |  |  |  | *JcbHLH88* | *VIT_18s0001g07410* |
|  |  |  |  |  |  | *JcbHLH89* | *VIT_18s0001g08040* |
|  |  |  |  |  |  | *JcbHLH90* | *VIT_18s0001g08600* |
|  |  |  |  |  |  | *JcbHLH91* | *VIT_18s0001g09210* |
|  |  |  |  |  |  | *JcbHLH92* | *VIT_18s0001g10270* |
|  |  |  |  |  |  | *JcbHLH94* | *VIT_18s0001g10400* |
|  |  |  |  |  |  | *JcbHLH95* | *VIT_17s0000g03580* |
|  |  |  |  |  |  | *JcbHLH98* | *VIT_04s0023g03430* |
|  |  |  |  |  |  | *JcbHLH101* | *VIT_14s0083g00930* |
|  |  |  |  |  |  | *JcbHLH102* | *VIT_14s0006g02850* |
|  |  |  |  |  |  | *JcbHLH104* | *VIT_02s0025g03450* |
|  |  |  |  |  |  | *JcbHLH106* | *VIT_02s0025g02610* |
|  |  |  |  |  |  | *JcbHLH107* | *VIT_13s0064g01290* |
|  |  |  |  |  |  | *JcbHLH107* | *VIT_06s0004g01740* |
|  |  |  |  |  |  | *JcbHLH110* | *VIT_03s0088g01240* |
|  |  |  |  |  |  | *JcbHLH112* | *VIT_03s0091g00730* |
|  |  |  |  |  |  | *JcbHLH114* | *VIT_13s0067g01350* |
|  |  |  |  |  |  | *JcbHLH116* | *VIT_11s0016g02070* |
|  |  |  |  |  |  | *JcbHLH117* | *VIT_11s0016g00380* |
|  |  |  |  |  |  | *JcbHLH120* | *VIT_11s0037g00040* |
